# Independence, institutionalization, death and treatment costs 18 months after rehabilitation of older people in two different primary health care settings

**DOI:** 10.1186/1472-6963-12-400

**Published:** 2012-11-14

**Authors:** Inger Johansen, Morten Lindbak, Johan K Stanghelle, Mette Brekke

**Affiliations:** 1 Department of General Practice/General Practice Research Unit, Institute of Health and Society, University of Oslo, PO Box 1130, Oslo, Blindern, N-0318, Norway; 2 Sunnaas Rehabilitation Hospital and Medical Faculty, University of Oslo, Oslo, Norway

**Keywords:** Aged and >80, Community rehabilitation, Activities of daily living, Costs

## Abstract

**Background:**

The optimal setting and content of primary health care rehabilitation of older people is not known. Our aim was to study independence, institutionalization, death and treatment costs 18 months after primary care rehabilitation of older people in two different settings.

**Methods:**

Eighteen months follow-up of an open, prospective study comparing the outcome of multi-disciplinary rehabilitation of older people, in a structured and intensive Primary care dedicated inpatient rehabilitation (PCDIR, n=202) versus a less structured and less intensive Primary care nursing home rehabilitation (PCNHR, n=100). Participants: 302 patients, disabled from stroke, hip-fracture, osteoarthritis and other chronic diseases, aged ≥65years, assessed to have a rehabilitation potential and being referred from general hospital or own residence. Outcome measures: Primary: Independence, assessed by Sunnaas ADL Index(SI). Secondary: Hospital and short-term nursing home length of stay (LOS); institutionalization, measured by institutional residence rate; death; and costs of rehabilitation and care. Statistical tests: T-tests, Correlation tests, Pearson’s χ^2^, ANCOVA, Regression and Kaplan-Meier analyses.

**Results:**

Overall SI scores were 26.1 (SD 7.2) compared to 27.0 (SD 5.7) at the end of rehabilitation, a statistically, but not clinically significant reduction (p=0.003 95%CI(0.3-1.5)). The PCDIR patients scored 2.2points higher in SI than the PCNHR patients, adjusted for age, gender, baseline MMSE and SI scores (p=0.003, 95%CI(0.8-3.7)). Out of 49 patients staying >28 days in short-term nursing homes, PCNHR-patients stayed significantly longer than PCDIR-patients (mean difference 104.9 days, 95%CI(0.28-209.6), p=0.05). The institutionalization increased in PCNHR (from 12%-28%, p=0.001), but not in PCDIR (from 16.9%-19.3%, p= 0.45). The overall one year mortality rate was 9.6%. Average costs were substantially higher for PCNHR versus PCDIR. The difference per patient was 3528€ for rehabilitation (p<0.001, 95%CI(2455–4756)), and 10134€ for the at-home care (p=0.002, 95%CI(4066–16202)). The total costs of rehabilitation and care were 18702€ (=1.6 times) higher for PCNHR than for PCDIR.

**Conclusions:**

At 18 months follow-up the PCDIR-patients maintained higher levels of independence, spent fewer days in short-term nursing homes, and did not increase the institutionalization compared to PCNHR. The costs of rehabilitation and care were substantially lower for PCDIR. More communities should consider adopting the PCDIR model.

**Trial registration:**

Clinicaltrials.gov ID NCT01457300

## Background

The main goal of rehabilitation is to achieve optimal functioning in interaction with the environment [1]. Older people express that their primary aim of rehabilitation after a disabling event is to return to their own residences and to live there as long as they wish with an optimal independence and quality of life [2,3]. To develop cost-effective rehabilitation systems is a growing challenge as the proportion of disabled older people is expected to increase substantially in developed countries in the coming decades [4,5]. The most common disabling conditions of older age are stroke and hip fracture, and more than half of the total health care costs of these conditions are related to long-term care [6,7].

Rehabilitation of older people is provided at the specialized-, intermediate- and primary health care level. At each level there are different rehabilitation programmes. Specialized- and intermediate rehabilitation can be inpatient, outpatient or home based and adapted for older patients with specific or different diagnoses. However, it is not clear if any of these programmes are cost-effective.

At the specialized level it has been shown that rehabilitation of older patients with different diagnoses in geriatric hospital departments improves function and reduces institutionalization and mortality, to a higher degree than in usual care [8]. Geriatric day hospital rehabilitation has also proven successful in terms of independence [9]. No difference in cost-effectiveness was found in a multi-centre RCT comparing rehabilitation of patients with different conditions in a geriatric hospital department with standard care [10]. Others have shown that the one year costs of medical care after intensive rehabilitation of patients with hip-fractures did not differ significantly from medical care after standard hospital rehabilitation [11]. An acute stroke-unit care combined with an Early Supported Discharge programme may reduce the length of hospital stay and improve independence without increasing the costs of outpatient rehabilitation compared with traditional stroke care [12]. Intermediate level services like community hospitals, Early Supported Discharge services and home based rehabilitation also report on gain in the level of independence of older patients with different conditions [9,13,14]. Post-acute treatment and rehabilitation of older patients in a community hospital were cheaper than rehabilitation in a general hospital, probably due to fewer readmissions [15]. A recent review paper concluded that programmes focusing on multi-disciplinary approach, accelerated rehabilitation and continuity of care, can reduce the care costs after hip-fractures [7].

However, there is little information about short- and long-term outcomes and costs of comprehensive primary health care rehabilitation of older people.

Due to the increased proportion of older people in the society, the shortage of hospital beds and a limited number of specialists in geriatric and rehabilitation medicine, it is important to study if a proportion of rehabilitation of older people can be managed successfully at the primary health care level. In a previous study we demonstrated that older patients disabled due to different conditions who received multi-disciplinary, structured and intensive rehabilitation in a primary health care inpatient dedicated rehabilitation centre (PCDIR) resulted in a higher level of independence within a shorter rehabilitation period as compared to standard primary health care rehabilitation in short-term beds in nursing homes (PCNHR). This difference sustained at three months follow-up [16,17]. In the present study we wanted to explore outcomes of the two rehabilitation models at 18 months follow-up.

### Aims

The primary aim of the study was to compare the level of independence of older patients 18 months after PCDIR and PCNHR and to study how this was influenced by patient characteristics, baseline diagnosis, cognitive and emotional status, and the duration and method of rehabilitation.

A secondary aim was to analyse hospital and short-term nursing home LOS, institutional residence rate and mortality during 18 months after the rehabilitation, and to examine how these variables were influenced by patient characteristics, baseline diagnosis, cognitive and emotional status, and the rehabilitation method.

A tertiary aim was to study the costs of rehabilitation and care in the two rehabilitation models.

## Methods

### Rehabilitation services for older people in Norway

In Norway the health care is mainly public and is divided into the specialized and the primary levels. Specialized rehabilitation services are provided both by the public and private health care, mainly in inpatient settings. From 2006 the private rehabilitation institutions have been partly funded through the public specialized health care system by a national agreement. The primary level rehabilitation services for older people are mainly provided in short-term beds in nursing homes, beds which are also intended to serve the relief-, palliative- and sub-acute care needs. Some municipalities have Home based rehabilitation served by multi-disciplinary ambulatory teams and some have dedicated inpatient facilities, as in the present study, but these services are in a minority. Like in other countries Norway has also through the last two decades developed some intermediate care rehabilitation services based on a shared care between the specialized and primary health care.

### Study design

This was an 18 months follow-up of an open, prospective comparative observational study.

### Setting

The study was carried out in two districts in the county of Vestfold, Norway. The number of inhabitants and the demographic, rural and urban distribution of people in the two districts were similar. In one district the multi-disciplinary primary care based rehabilitation of older patients was provided in a dedicated inpatient centre (PCDIR ), and in the other district in short-term beds in nursing homes (PCNHR). The key features of the setting and content of the two rehabilitation models are shown in Table 1, which is a modification of a more extensive table published elsewhere [17]. The PCDIR study centre has 16 beds and covers a population of 40.000 inhabitants. It is a completely free-standing facility. The patients pay out of pocket 130NOK (=16€) per day for this care, which is based on a national agreement for services in all Norwegian primary care short-term institutions. The centre has a 50% part-time general practitioner involvement, full-time four physio- and three occupational therapists, in addition to the nursing care personnel. The assessment, rehabilitation process and focus in the PCDIR centre is very similar to the essential elements of successful rehabilitation described in the WHO rehabilitation cycle [1]. The recruitment period was from June 2006 until April 2009. The exposure time was the rehabilitation period. In our previous studies we looked at data collected at the beginning, two weeks into, at completion of and three months after the rehabilitation. In the present study we collected data at 18 months after the rehabilitation. Data were collected by the first author, by qualified personnel in the rehabilitation centre and by two project assistants. The first author coordinated the data collection.

**Table 1:** Main characteristics of Primary Care Dedicated Inpatient Rehabilitation and Primary Care Nursing Home Rehabilitation

**Rehabilitation feature**	**PCDIR**^**1**^	**PCNHR**^**2**^
Multi-dimensional assessment	Standardized	Not standardized
Professionals of the rehabilitation team	GP, nurse, physio- and occupational therapist. Other professionals at need	GP, nurse, physio- and occupational therapist. Other professionals at need
Rehabilitation arena	Short term beds in primary care dedicated inpatient rehabilitation centre	Short-term beds in primary care nursing homes
Focus of the setting	Continuous rehabilitation focus in an optimistic and realistic setting	Frequent shift of focus between rehabilitation and care
Rehabilitation process		
Goals, plan, intervention tailored to the patient	Always	Occasional
Measurement instruments	Always, 3-4 regular	Occasional
Collaboration between patient, staff, relatives and primary health care	Close, in at least weekly meetings	Occasional
Training: Physical-, functional-, ADL-	In groups, one-by-one and self-training	In groups, one-by-one and self-training
Training intensity/frequency	Three hours/day	Two hours/day

PCDIR= Primary Care Dedicated Inpatient Rehabilitation 2. PCNHR= Primary Care Nursing Home Rehabilitation.

### Participants

The study population was disabled older people living in the two districts described above. They were admitted to rehabilitation either post-acute from the district general hospital or from their own residences. Inclusion criteria were both genders and age ≥ 65years. The referral diagnoses were disability due to stroke, osteoarthritis, hip fracture and “others” (ageing disability, loss of function due to long periods of hospitalization and chronic, slowly progressing diseases). Only patients considered to have a rehabilitation potential were included. Rehabilitation potential was defined as the physiological and psychological possibilities of a disabled patient to restore, improve or maintain an optimal level of function and quality of life [6]. Assessment of the rehabilitation potential was based on a total evaluation of the level of ADL, cognitive, emotional and physical function, as well as the patient’s motivation to an active rehabilitation process. The assessment was made by a multi-disciplinary team and in the same way for all patients. Details as to the minimum required ADL and cognitive levels are described in the section “Variables and outcome measurements”. Patients with active psychoses or severe depressions with a lack of initiative were not included. Other exclusion criteria were patients with rapidly progressive diseases, severe chronic obstructive pulmonary disease, unstable angina pectoris and not clarified cardiac arythmias. Patients were included consecutively upon admission to rehabilitation. Approximately half of the patients in both models were admitted from the district general hospital and the other half directly from their own residences. The recruitment process is fully described in a previous paper [17].

### Variables and outcome measurements

The validated scale Sunnaas ADL Index, SI [18] was the main outcome measure and indicator of independence. SI measures 12 activities of daily life. Each activity has a score from 0–3, where 0=totally dependent and 3=independent. The total maximum score of 36 means totally independent. Scores <12 means that the patient needs help from one or more persons in nearly all ADL situations, which in most cases indicate a marginal rehabilitation potential. The majority of the study patients had baseline SI scores from 20–25.

MMSE, Mini Mental Status Evaluation [19], measures cognitive function, which was considered a possible predictor of outcome. Scores are from 0–30. Patients with hip-fracture and mild (MMSE score 18–23) or moderate (MMSE score 12–17) dementia can often return to the community if they are provided with active geriatric rehabilitation [20,21]. In our study we did not consider patients to have a rehabilitation potential if the MMSE scores were <18-20, but if the pre-rehabilitation motor ability was good, they were included. MMSE was recorded two weeks into the rehabilitation to avoid recording incidental confusion at baseline.

SCL-10, Symptom Check List-10 [22] is a validated questionnaire mapping emotional health during the previous week, particularly anxiety and depression, and was included as a possible predictor of outcome. SCL-10 comprises ten questions with scores from 1–4. The final score is the total score sum divided by ten. Scores>1.85 indicate severe emotional problems. SCL-10 was recorded two weeks into rehabilitation to avoid recording possible emotional instability at baseline.

Other secondary outcome variables were hospital and short-term nursing home LOS, institutionalization as measured by institutional residence rate, and mortality during 18 months after the rehabilitation. The source of this information was the GP- and nursing care files of the patients and the official Norwegian Death Registry.

Age, gender, marital status and diagnostic group were recorded at baseline. Type of residence was recorded at baseline and at 18 months follow-up.

### Cost calculations

Cost calculations were based on average costs per patient according to the 2009 price level. (8 Norwegian kroner (NOK)=1 Euro(€)). The per patient PCDIR costs (2750NOK=343€/day) and the hospital costs (4000NOK=500€/day) were given from the official accounts of the specific institutions. The per patient nursing home costs (2280NOK=285€/day) were given from Statistics Norway [23]. The per patient costs/hour of at-home care (624NOK=78€/hour) were calculated from data provided by the community administrations of the study districts and were based on the average costs of nursing, care, utensils, transportation and administration. The level of at-home care (hours/day) was recorded in the previous studies at end of and three months after the rehabilitation [17]. These levels correlated very strongly to the corresponding SI scores (PCDIR:-0.7 and PCNHR:-0.9 (p>0.001), Pearson’s correlation coefficient). Based on this very strong correlation, the fact that there was no clinically significant change in SI scores during the 18months follow-up, and that the difference in SI scores between the two models sustained (Result section, present paper), we calculated that the level of at-home care services followed the same pattern as the SI scores during the 18 months follow-up.

### Sample size

A two points difference in SI between the two models was judged to be clinically significant. Power calculation estimated a need for including 100 patients in each model, based on a beta of 0.90, an alpha of < 0.05 and SD=4.3 in SI. We decided to include 200 patients in PCDIR to ensure enough patients for subgroup analyses [16].

### Statistics

Data were analyzed in SPSS version 19.0 for Windows. Two groups of continuous, symmetrically distributed variables were compared by T-tests, and several groups by one way ANOVA (posthoc test if p<0.05). Asymmetrically continuous variables were compared by Mann–Whitney Wilcoxon-test. Correlations between continuous variables were analysed by Pearson’s (symmetrical distribution) or Spearman’s (asymmetrical distribution) correlation coefficient. Categorical variables were compared by Pearson’s χ^2^ test. Differences in SI gain between the groups were analysed by ANCOVA (Analysis of covariance) to correct for SI imbalance at baseline [24]. Possible predictors of outcome were identified by univariate regression analysis, and statistically significant variables were analysed in multiple linear regression analysis to identify confounders and true predictors. Survival was analysed by Kaplan-Meier analysis.

### Ethics

The study was approved by the Regional Ethics Committee for Medical Research and by the Norwegian Social Science Data Services.

The study clinicaltrials.gov ID is NCT01457300.

## Results

### Participants

In total 302 patients were recruited into the study at baseline, 202 into PCDIR and 100 into PCNHR. Eligible patients were recruited consecutively throughout the recruitment period. All eligible patients were asked and all but one accepted and gave informed consent to participate in the study. Consent was given on admission to the rehabilitation. Two of the patients in PCDIR were excluded shortly after inclusion due to a serious stroke and a leg amputation, respectively. Totally 43 patients died during the 18 months follow-up period, and two patients were lost to follow-up, which left 255 patients for follow-up assessment at 18 months.

### Descriptive data

Table 2 shows patient characteristics, diagnoses and baseline cognitive and emotional status of the total study population and the PCDIR and PCNHR populations surviving at 18 months follow-up. The women in both models were older than the men and more frequently lived alone and suffered from hip fracture. The men more often suffered from stroke.

**Table 2:** Characteristics of older patients surviving at 18 months after primary care inpatient rehabilitation

	**Total population**	**PCDIR**^**1**^	**PCNHR**^**2**^
Number of patients (n)	255	166	89
Age y mean (SD, min-max)	81.7 (6.8, 65-96)	81.8 (5.9, 66-95)	81.5 (6.6, 66-96)
Gender men/women (n)	74/181	45/121	29/60
Residence (N=254)			
Own (n)	198 (78%)	134 (81%)	64 (72%)
Care-flat/long term nursing home(n)	56 (22%)	32 (19%)	25 (28%)
Marital status: Married (n)	98 (38%)	60 (36%)	38 (43%)
Alone (n)	157 (62%)	106 (64%)	51 (57%)
Diagnoses (N=254)			
Stroke (n)	43 (17%)	30 (18%)	13 (15%)
Osteoarthritis (n)	34 (13%)	20 (12%)	14 (16%)
Fracture (n)	92 (36%)	66 (40%)	26 (29%)
Other (n)	85 (34%)	49 (30%)	36 (40%)
MMSE^3^, mean (SD)	25.3 (3.9)	25.2	25.3
(CI) (N=255)	(24.8-25.8)		
SCL10^4^, mean (SD)	1.4 (0.3)	1.4	1.4
(CI) (N=255)	(1.3-1.4)		
Men/women			
Age, years	79.7/82.5^5^		
Living alone, %	36/72^6^		
Fracture, %	27/40^6^		
Stroke, %	30/12^6^		
1. PCDIR=Primary Care Dedicated Inpatient Rehabilitation			
2. PCNHR=Primary Care Nursing Home Rehabilitation			
3. MMSE=Mini Mental Status Evaluation			
4. SCL10=Symptom Checklist 10			
5. Independent Samples T-test, p<0.001 95% CI(1.0-4.7)			
6. Pearson χ ^2^ p=0.002			

### Level of and predictors for ADL-function at 18 months follow-up

The patients scored 26.1 (SD 7.2) points in SI at 18 months compared to 27.0 (SD 5.7) points at end of the rehabilitation period, a statistically, but not clinically significant reduction of 0.9 point (p=0.003 95%CI(0.3-1.5), Paired Samples T-T).

The predictor analyses showed that SI at 18 months follow-up was independent of gender, marital status, diagnoses, emotional status and the duration of the rehabilitation and was predicted by age, cognitive status and the rehabilitation method. The exact results were that if other variables were kept constant, a one year higher age meant a 0.1 point lower level of SI, a one point higher MMSE score meant a 0.5 point higher level of SI and a change in rehabilitation method from PCNHR to PCDIR meant a 2.2 points higher level of SI [Table 3].

**Table 3:** **Predictors of independence**^**1**^**at 18 months after primary care inpatient rehabilitation of older people**

	**USB**^**2**^	**p**	**95% CI of B**
Constant	13.5	.013	
Gender	-.7	.376	−2.2-.8
Age	-.1	.040	-.2--.005
SI baseline	.5	<.001	.4-.6
MMSE	.5	<.001	.3-.7
Rehabilitation Method	2.2	.003	.8-3.7

Dependent variable SI 18 months after the rehabilitation.

1 Independence=Ability to perform activities of daily living=Sunnaas ADL Index=SI.

2 USB=Ustandardized Coefficient B in a Multiple linear regression analysis, corrected for gender, age and SI at baseline, shows the change in SI at 18 months after rehabilitation (USB) when the variable changes one point.

### Short-term nursing home and hospital LOS, institutionalization and death until 18 months follow-up

Ninety-four (37%) of the patients had short-term nursing home stays, and the patients in PCNHR had longer LOS compared to PCDIR (Table 4). Sixty six (26%) of the patients had hospital stays, mean 16.1 days in PCDIR (n=41), and 9.6days in PCNHR (n=25). The difference was not statistically significant (p=0.1 Independent Samples T-test).

**Table 4:** Mean days in short-term nursing homes from 0–18 months after primary care inpatient rehabilitation of older people in two different settings

	**PCDIR**^**1**^	**PCNHR**^**2**^	**Difference mean (95%CI)**^**3**^	**P of the difference**^**3**^
Days in short-term nursing homes				
0 days	n=102(62%)	n=58(66%)		
1-28 days, Mean				
(95%CI)	16.9(15.1-18.8)	20.5(16.8-24.1)	3.6(-7.1-0.1)	0.06
	(n=32, 19%)	(n=13, 15%)		
>28 days,				
Mean(95%CI)	148.5(91.7-205.6)	253.6(150.8-356.4)	104.9(0.28-209.6)	0.05
	(n=32, 19%)	(n=17, 19%)		

1 Primary Care Dedicated Inpatient Rehabilitation.

2 Primary Care Nursing Home Rehabilitation.

3 Independent Samples T-test.

Sixteen (11.8%) of the patients aged ≥80years resided in a nursing home at 18 months follow-up (9 (9.8%) in PCDIR and 7(15.6%) in PCNHR), compared to no patients at baseline. The proportion of patients residing in a care-flat or nursing home increased significantly in PCNHR, (from 12(12.0%) to 25(28.1%) (McNemar, p=0.001)), but not in PCDIR (from 28(16.9%) to 32(19.3%) (McNemar p=0.45)).

**Figure 1:**
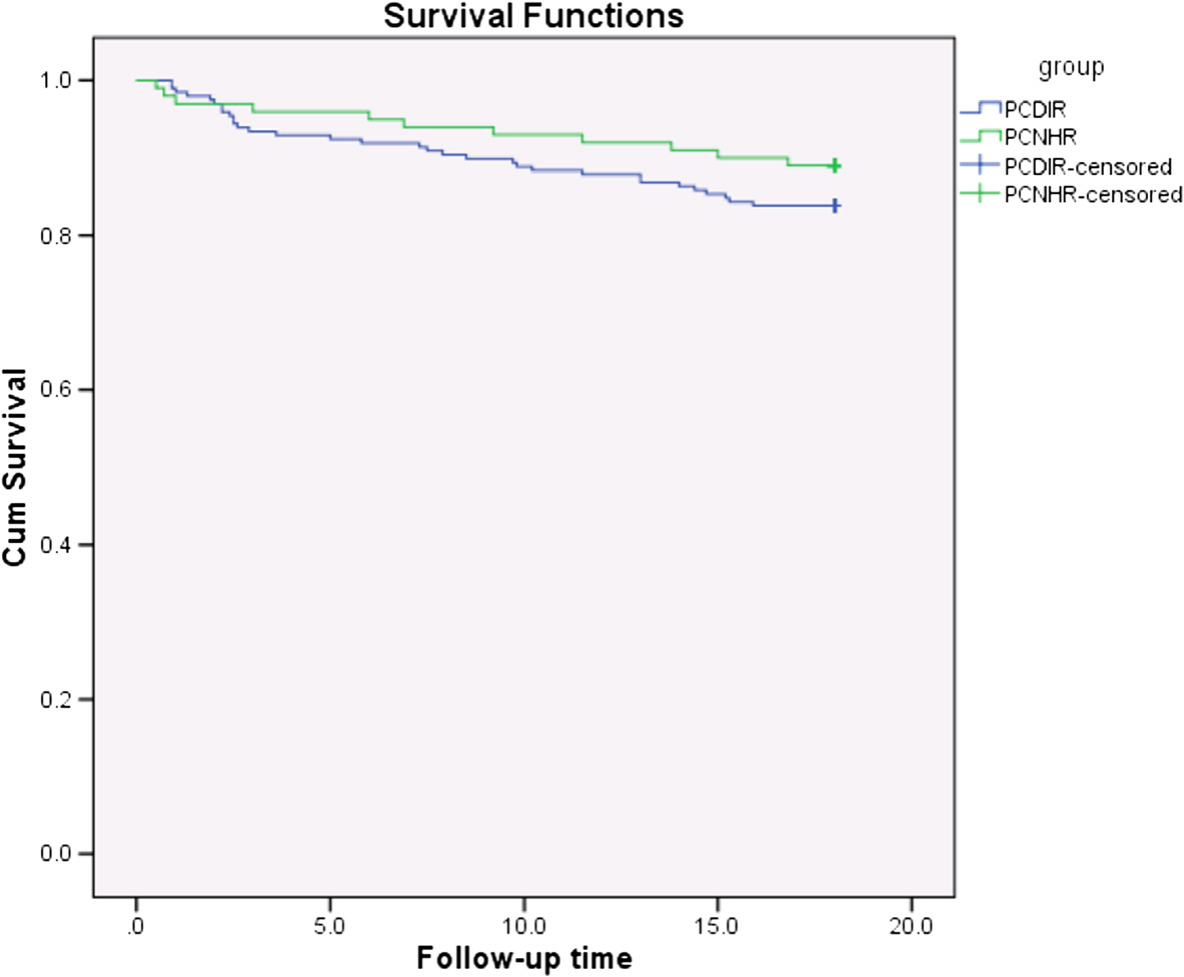
**Survival curves(Kaplan-Meier) for older patients 0–18 months after primary care rehabilitation in two different settings.** Log Rank (Mantel-Cox) χ^2^ Test P=0.23. PCDIR: Primary Care Dedicated Inpatient Rehabilitation PCNHR: Primary Care Nursing Home Rehabilitation Follow-up time in months.

Forty-three of the 298 patients (Excluded=2, Lost to follow-up=2) died during the study period, giving a one year mortality of 9.6%. The patients who died were older than the surviving patients and had lower SI at the beginning and end of the rehabilitation period, (age at baseline 82.9 versus 80.2 years, p=0.01, 95%CI(0.5-4.9), SI at beginning: 20.9 versus 23.3, p=0.04 95%CI(0.1-4.7), SI at end: 24.4 versus 27.0, p=0.03 95%CI(0.2-5.0) - Independent Samples T-test). The difference in survival curves for the patients in the two rehabilitation models was not statistically significant (Figure 1).

### Predictors of number of days in nursing homes

The predictor analyses showed that the number of short-term days in nursing homes were independent of gender, age, marital status, emotional status, diagnoses and the rehabilitation method and predicted negatively by cognitive status and SI at end of rehabilitation. The exact results were that if other variables were kept constant, a one point higher MMSE score meant 6.4 fewer days in nursing homes (p<0.001 95%CI(−3.1—9.7)) and a one point higher SI at end of rehabilitation meant 5.4 fewer days in nursing homes (p<0.001 95%CI(−3.1—7.7)).

### Rehabilitation and care costs

The average rehabilitation costs were 3 528€ higher per patient in PCNHR compared to PCDIR and the at-home care costs were 10 134€ higher. Both differences were statistically significant (Table 5). The mean costs of nursing home care per patient staying >28 days, which included 19% of the patients in both models, were 29 897€ higher in PCNHR compared to PCDIR, a statistically significant difference (Table 5). The average total costs per patient for rehabilitation, at-home, hospital and short-term nursing home care were 48 147€ in PCNHR (Table 5), which was 1.6 times higher compared to in PCDIR.

**Table 5:** **Rehabilitation and care costs**^**1**^**per patient during 18 months after primary care inpatient rehabilitation of older people**

**Setting**	**PCDIR**^**2**^**mean(CI)**	**PCNHR**^**3**^**mean(CI)**	**Cost difference (PCNHR-PCDIR) mean (CI)**^**4**^	**p**^**4**^**of the cost difference**
Rehabilitation^5^	7 443	10 972	3 528	<0.001
	(6 963–7 923)	(9 376–12 369)	(2 455–4 756)	
At-home Care^6^	10 890	20 995	10 134	0.002
	(10 221–11 772)	(18 689–23 301)	(4 066–16 202)	
Hospital^6^	2 020	1 360	−660	0.3
	(1 180–2 865)	(550–2 165)	(−600-1 950)	
Nursing home total^6^	9 092	14 820	5 728	0.2
	(5 301–13 253)	(6 897–22 743)	(−3 078–14 505)	
Sum rehabilitation and care	29 445	48 147	18 702	
Nursing home 0 days	0 (n=102, 62%)	0 (n=58, 66%)	0	
Nursing home 1–28 days	4 817	5 843	1 026	0.06
	(4 304–5 358)	(4 788–6 869)	(−29-2 021)	
	(n=32, 19%)	(n=13, 15%)		
Nursing home >28 days	42 380	72 276	29 897	0.05
	(26 135–58 596)	(42 978–101574)	(80–59 736)	
	(n=32, 19%)	(n=17, 19%)		

1 Costs in €(2009 price level, 1€=8NOK).

2 Primary Care Dedicated Inpatient Rehabilitation.

3 Primary Care Nursing Home Rehabilitation.

4 Independent Samples T-test.

5 PCDIR: n=200, PCNHR: n=100.

6 PCDIR: n=166, PCNHR: n=89.

## Discussion

This study showed that disabled older patients who received multi-disciplinary PCDIR maintained a statistically and clinically significant higher level of independence from end of rehabilitation until 18 months afterwards, spent fewer days in short-term nursing homes and did not increase the institutional residence rate, compared to patients who received PCNHR. The rehabilitation and care costs of PCDIR were substantially lower.

Irrespective of the type of rehabilitation, cognitive status was a predictor of both the level of independence and the number of short-term days in nursing homes. This is consistent with our previous findings at end of and three months after the rehabilitation [17]. According to our experience the ability of initiative and to take instructions were the cognitive features of greatest importance for successful rehabilitation. Several studies identify cognitive status as a predictor of rehabilitation outcomes [25,26].

Due to the disability of the study population, we expected that the institutional residence rate at 18 months follow-up would be higher than in the general Norwegian population at the same age. However, while 9.8% of the PCDIR and 15.6% of the PCNHR patients ≥80 years lived in nursing homes, 14.3% of the general Norwegian population of the same age group resided in nursing homes in 2007 [27]. Our data indicate that PCDIR, if adopted on a broader scale, may reduce the number of Norwegians ≥80 years living in nursing homes (in 2007 n=31.000) by several thousands.

The one year mortality of the total study population was higher than in the general Norwegian population at the same age, 9.6% versus 6%, respectively [28]. Mortality rates reported after post-acute rehabilitation of older people are about 20% [29,30]. Only half of the patients in our study were in post-acute rehabilitation, which may explain some of the difference. Furthermore, the major causes of death in post-acute rehabilitation and care studies are cardiovascular, infectious and malignant diseases. Only a few patients with these diagnoses were included in our study [17]. Due to their higher ADL levels, we expected the PCDIR patients to have a better survival than the PCNHR patients. Surprisingly, there was a not statistically significant tendency towards the opposite. This may be explained by the higher morbidity as shown by more days in hospital.

The PCDIR intervention in this study was both more effective and less expensive compared to the PCNHR, thus meeting the criteria for a preferred strategy [7]. In such cases the health-care decisions are obvious and calculation of a cost-effective ratio is not necessary. The main reasons for the lower costs of the PCDIR were the shorter rehabilitation stay and the lower at-home care needs compared to the PCNHR. The costs of medication, transportation and outpatient physician and physiotherapy visits were not recorded, but we could not give any reasons that these costs would influence the cost differences in our study. The average total costs per patient were 1.6 times higher in PCNHR during 18 months follow-up. However, if further survival time is taken into account, the cost differences might be even higher. The remaining life time of 82 years old Norwegians is about seven years (men: six years, women: eight years) [31].

A limitation to the study was the non-randomized design. We wanted to perform a study of the “real-life health care”, and a study of level 2 design was our nearest option to achieve more knowledge about this important and poorly investigated field. The first author worked as a GP in the rehabilitation centre when the PCDIR patients were recruited, which could have introduced a bias. She did the general clinical evaluation of the patients, but was not involved in the training of the patients and did none of the SI scores. Methodical weaknesses have been thoroughly discussed in a previous paper [17]. On the other hand, patient features likely to influence the outcomes were not different in the two rehabilitation models [Table 2, and all participants were considered to have a rehabilitation potential, which was assessed in the same way in the two models. Most of the procedures and decisions were standardized.

The measurement scales used in this study are proven to be valid, reliable and sensitive to change over time. SI is not widely used internationally, but it is the commonly used ADL-scale in primary care in the study county. The inter-item consistency between the internationally commonly used FIM and SI is high for many items, even if differences also exist [32]. We believe that when clinically significant improvements in different ADL-scales are defined, it is possible to compare different ADL-scales in terms of level of independence.

We have not found other studies evaluating the long-term outcomes of a dedicated primary health care based rehabilitation similar to the present model. However, both intermediate and specialized multi-disciplinary, inpatient rehabilitation of older people have shown a benefit in long-term (3-12months) outcomes compared to standard community or general hospital care [8,29,33,34]. Studies of these rehabilitation programmes for older people in general-, orthopedic- and stroke rehabilitation report higher long-term levels of independence [8,29,33-35] and lower long-term levels of institutionalization [8,33,35] and mortality [8,29,35,36]. More intensive exercise increases the success of hip-fracture programmes [37,38].

Cost-saving effects of different rehabilitation strategies are unclear, and it is difficult to compare costs across countries since both the reimbursement systems, delivery agreements and the price levels differ. Norwegian community hospitals are likely to provide health care at lower costs than alternative models of care, like general hospitals, nursing homes and at-home care [39]. A community hospital in the Netherlands was also shown to be a cost-saving alternative for older patients in need of intermediate medical and nursing home care between hospital and at-home care [40]. The lower one year costs of a Norwegian post-acute community hospital compared to a general hospital might be out-weighed by a higher proportion of the patients residing in a nursing home at follow-up [15]. Sub-acute nursing homes were more effective than traditional nursing homes in returning patients aged ≥65years with stroke to the community, but the Medicare costs were greater [41].

The PCDIR model includes the main features of the WHO rehabilitation cycle [1]. We believe that rehabilitation programmes which adhere to this cycle are more likely to be beneficial [8].

## Conclusions

This study shows that disabled older people who receive multi-disciplinary PCDIR, maintain higher levels of independence, spend fewer days in short-term nursing homes and do not have increased institutionalization during 18 months follow-up, compared to disabled older people who receive multi-disciplinary PCNHR. The PCDIR model is shown to be both more effective and less expensive. To sustain independence and reduce institutionalization and treatment costs among older people, more communities should consider adopting the PCDIR model, which includes the main features of the WHO rehabilitation cycle, into the primary health care.

## Abbreviations

PCDIR: Primary Care Dedicated Inpatient Rehabilitation; PCNHR: Primary Care Nursing Home Rehabilitation; SI: Sunnaas ADL Index; MMSE: Mini Mental Status Evaluation; SCL10: Symptom Checklist 10; LOS: Length of stay.

## Competing interests

The authors declare that they have no competing interests.

## Authors’ contributions

IJ conceived the study and contributed substantially to its design and the collection of data. She performed the statistical analyses, interpreted the data and drafted the manuscript in collaboration with MB, who was the main supervisor. IJ wrote the paper. MB, ML and JKS contributed substantially to the design of the study. ML and JKS participated in the interpretation of the data and revised the manuscript critically for important intellectual content. All authors read and approved the final manuscript.

## Pre-publication history

The pre-publication history for this paper can be accessed here:

http://www.biomedcentral.com/1472-6963/12/400/prepub

## References

[B1] Stucki G Cieza A Melvin J The international classification of functioning, disability and health (ICF): a unifying model for the conceptual description of the rehabilitation strategy J Rehabil Med 2007 39 279 285 10.2340/16501977-0041 17468799

[B2] Gillsjo C Schwartz-Barcott D von P I: Home: the place the older adult cannot imagine living without BMC Geriatr 2011 11 10 10.1186/1471-2318-11-10 21410994 PMC3072327

[B3] Fange A Ivanoff SD The home is the hub of health in very old age: findings from the ENABLE-AGE project Arch Gerontol Geriatr 2009 48 340 345 10.1016/j.archger.2008.02.015 18423909

[B4] WHO Active ageing: Towards age-friendly primary health care 2004

[B5] Jagger C Mattews R Spiers N Brayne C Comas-Herrera A Robinson T Lindesay J Croft P Compression or Expansion of Disability? Forecasting lFuture Disability Levels Under Changing Patterns of Diseases. Final report 2006 University of Leicester KF 117 02/06

[B6] Norwegian government’s white paper “Responsibility and empowerment” 1998

[B7] Haentjens P Lamraski G Boonen S Costs and consequences of hip fracture occurrence in old age: an economic perspective Disabil Rehabil 2005 27 18–19 1129 1141 16278182 10.1080/09638280500055529

[B8] Bachmann S Finger C Huss A Egger M Stuck AE Clough-Gorr KM Inpatient rehabilitation specifically designed for geriatric patients: systematic review and meta-analysis of randomized controlled trials BMJ 2010 340 c1718 10.1136/bmj.c1718 20406866 PMC2857746

[B9] Forster A Young J Community rehabilitation for older people: day hospital or home-based services? Age Ageing 2010 0 1 3 10.1093/ageing/afq136 21098621

[B10] Kehusmaa S Autti-Ramo I Valaste M Hinkka K Rissanen P Economic evaluation of a geriatric rehabilitation programme: a randomized controlled trial J Rehabil Med 2010 42 10 949 955 10.2340/16501977-0623 21031292

[B11] Huusko TM Karppi P Avikainen V Kautiainen H Sulkava R Intensive geriatric rehabilitation of hip fracture patients: a randomized, controlled trial Acta Orthop Scand 2002 73 4 425 431 10.1080/00016470216324 12358116

[B12] Fjaertoft H Indredavik B Magnussen J Johnsen R Early supported discharge for stroke patients improves clinical outcome. Does it also reduce use of health services and costs? One-year follow-up of a randomized controlled trial Cerebrovasc Dis 2005 19 6 376 383 10.1159/000085543 15860914

[B13] Garasen H Windspoll R Johnsen R Intermediate care at a community hospital as an alternative to prolonged general hospital care for elderly patients: a randomised controlled trial BMC Public Health 2007 7 68 10.1186/1471-2458-7-68 17475006 PMC1868721

[B14] Fjaertoft H Indredavik B Lydersen S Stroke unit care combined with early supported discharge: long-term follow-up of a randomized controlled trial Stroke 2003 34 11 2687 2691 10.1161/01.STR.0000095189.21659.4F 14576376

[B15] Garasen H Magnussen J Windspoll R Johnsen R [Elderly patients in hospital or in an intermediate nursing home department--cost analysis] Tidsskr Nor Laegeforen 2008 128 3 283 285 18264150

[B16] Johansen I Lindbaek M Stanghelle JK Brekke M Effective rehabilitation of older people in a district rehabilitation center J Rehabil Med 2011 43 5 461 464 10.2340/16501977-0792 21390482

[B17] Johansen I Lindbaek M Stanghelle JK Brekke M Structured community-based inpatient rehabilitation of older patients is better than standard primary health care rehabilitation – an open comparative study Disabil Rehabil 2012 1 8 Early online 10.3109/09638288.2012.667193 22452632

[B18] Bathen T Vardeberg K Test-retest Reliability of the Sunnaas ADL Index Scand J Occup Ther 2001 8 140 147 10.1080/110381201750464494

[B19] Engedal K Haugen PK Gilje K Laake P Efficacy of short mental tests in the detection of mental impairment in old age Compr Gerontol A 1988 2 87 93 3228822

[B20] Beloosesky Y Grinblat J Epelboym B Weiss A Grosman B Hendel D Functional gain of hip fracture patients in different cognitive and functional groups Clin Rehabil 2002 16 321 328 10.1191/0269215502cr497oa 12017519

[B21] Huusko TM Karppi P Avikainen V Kautiainen H Sulkava R Randomised, clinically controlled trial of intensive geriatric rehabilitation in patients with hip fracture: subgroup analysis of patients with dementia BMJ 2000 321 1107 1111 10.1136/bmj.321.7269.1107 11061730 PMC27517

[B22] Strand BH Dalgard OS Tambs K Rognerud M Measuring the mental health status of the Norwegian population: a comparison of the instruments SCL-25, SCL-10, SCL-5 and MHI-5 (SF-36) Nord J Psychiatry 2003 57 113 118 10.1080/08039480310000932 12745773

[B23] Statistics Norway http://statbank.ssb.no/statistikkbanken/Default_FR.asp?PXSid=0&nvl=true&PLanguage=0&tilside=selectvarval/define.asp&Tabellid=07790

[B24] Vickers AJ Altman DG Analysing controlled trials with baseline and follow up measurements BMJ 2001 323 1123 1124 10.1136/bmj.323.7321.1123 11701584 PMC1121605

[B25] Cameron ID Schaafsma FG Wilson S Baker W Buckley S Outcomes of rehabilitation in older people–functioning and cognition are the most important predictors: an inception cohort study J Rehabil Med 2012 44 1 24 30 10.2340/16501977-0901 22124759

[B26] Poynter L Kwan J Sayer AA Vassallo M Does cognitive impairment affect rehabilitation outcome? J Am Geriatr Soc 2011 59 11 2108 2111 10.1111/j.1532-5415.2011.03658.x 22092047

[B27] Statistics Norwayhttp://www.ssb.no/00/01/20/valgaktuelt/arkiv/art-2007-08-28-01.html

[B28] Statistics Norwaywww.ssb.no, dra@ssb.no

[B29] Garåsen H Windspoll R Johnsen R Long-term patients’ outcome after intermediate care at a community hospital for elderly patients: 12 months follow-up of a randomized controlled trial Scan J Public Health 2008 36 2 197 204 10.1177/1403494808089685 18519285

[B30] Baztán JJ Gálvez CP Socorro A Recovery of functional impairment after acute illness and mortality: One-year follow-Up study Gerontology 2009 55 269 274 10.1159/000193068 19141990

[B31] Statistics Norway http://statbank.ssb.no/statistikkbanken/Default_FR.asp?PXSid=0&nvl=true&PLanguage=0&tilside=selectvarval/define.asp&Tabellid=05375

[B32] Claesson L Svensson E Measures of order consistency between paired ordinal data: application to the functional independence measure and sunnaas index of ADL J Rehabil Med 2001 33 137 144 10.1080/165019701750166014 11482355

[B33] Beswick AD Rees K Dieppe P Ayis S Gooberman-Hill R Horwood J Complex interventions to improve physical function and maintain independent living in elderly people: a systematic review and meta-analysis Lancet 2008 371 9614 725 735 10.1016/S0140-6736(08)60342-6 18313501 PMC2262920

[B34] Young J Green J Forster A Small N Lowson K Bogle S George J Heseltine D Jayasuriya T Rowe J Postacute care for older people in community hospitals: a multicenter randomized, controlled trial J Am Geriatr Soc 2007 55 12 1995 2002 10.1111/j.1532-5415.2007.01456.x 17979957

[B35] Fjaertoft H Rohweder G Indredavik B Stroke unit care combined with early supported discharge improves 5-year outcome: a randomized controlled trial Stroke 2011 42 6 1707 1711 10.1161/STROKEAHA.110.601153 21474806

[B36] Chen LK Chen YM Hwang SJ Peng LN Lin MH Lee WJ Effectiveness of community hospital-based post-acute care on functional recovery and 12-month mortality in older patients: a prospective cohort study Ann Med 2010 42 8 630 636 10.3109/07853890.2010.521763 20883138

[B37] Handoll HH Cameron ID Mak JC Finnegan TP Multidisciplinary rehabilitation for older people with hip fractures Cochrane Database Syst Rev 2009 4 CD007125 10.1002/14651858.CD007125.pub2 19821396

[B38] Stott D Handoll H Rehabilitation of older people after hip (proximal femoral) fracture Cochrane Database Syst Rev 2011 8 ED000023 10.1002/14651858.ED000023 21833982 PMC10846457

[B39] Aaraas I Sorasdekkan H Kristiansen IS Are general practitioner hospitals cost-saving? evidence from a rural area of Norway Fam Pract 1997 14 5 397 402 10.1093/fampra/14.5.397 9472375

[B40] Hakaart-van Roijen L van Charante EP M Bindels PJ A cost study of a general practitioner hospital in the Netherlands Eur J Gen Pract 2004 10 45 49 10.3109/13814780409094231 15232523

[B41] Kramer AM Steiner JF Schlenker RE Eilertsen TB Hrincevich CA Tropea DA Outcomes and costs after hip fracture and stroke. a comparison of rehabilitation settings JAMA 1997 277 5 396 404 10.1001/jama.1997.03540290048031 9010172

